# Supplementary material for: Self-Assembly by Tridentate or Bidentate Ligand: Synthesis and Vapor Adsorption Properties of Cu(II), Zn(II), Hg(II) and Cd(II) Complexes Derived from a Bis(pyridylhydrazone) Compound
Source: Molecules. 2020 Dec 29;26(1):109. doi: 10.3390/molecules26010109 (PMC7795747; doi:10.3390/molecules26010109)

checkCIF/PLATON report

Structure factors have been supplied for datablock(s) qq

THIS REPORT IS FOR GUIDANCE ONLY. IF USED AS PART OF A REVIEW PROCEDURE FOR PUBLICATION, IT SHOULD NOT REPLACE THE EXPERTISE OF AN EXPERIENCED CRYSTALLOGRAPHIC REFEREE.

No syntax errors found. CIF dictionary Interpreting this report

Datablock: qq

Bond precision: C-C = 0.0136 A Wavelength=0.71073

Cell:	a=10.227(6)	b=11.498(7)	c=13.310(8)
	alpha=113.636(19)	beta=90.801(19)	gamma=102.829(19)
Temperature:	273 K		

	Calculated	Reported
Volume	1388.7(15)	1388.7(15)
Space group	P -1	P -1
Hall group	-P 1	-P 1
Moiety formula	C52 H56 Cu4 N12 O10 [+ solvent]	C52 H56 Cu4 N12 O10
Sum formula	C52 H56 Cu4 N12 O10 [+ solvent]	C52 H56 Cu4 N12 O10
Mr	1263.29	1263.24
Dx,g cm-3	1.511	1.511
Z	1	1
Mu (mm-1)	1.578	1.578
F000	648.0	648.0
F000’	649.48	
h,k,lmax	11,13,15	11,13,15
Nref	4373	4358
Tmin,Tmax	0.705,0.777	0.578,0.746
Tmin’	0.689	

Correction method= # Reported T Limits: Tmin=0.578 Tmax=0.746
AbsCorr = NONE

Data completeness= 0.997 Theta(max)= 24.000

R(reflections)= 0.0705(2454) wR2(reflections)= 0.2020(4358)

S = 1.042 Npar= 361

The following ALERTS were generated. Each ALERT has the format
test-name_ALERT_alert-type_alert-level.
Click on the hyperlinks for more details of the test.

Alert level B

THETM01_ALERT_3_B The value of sine(theta_max)/wavelength is less than 0.575
Calculated sin(theta_max)/wavelength = 0.5723
PLAT031_ALERT_4_B Refined Extinction Parameter Within Range ...... 1.833 Sigma
PLAT234_ALERT_4_B Large Hirshfeld Difference O5 --C26 . 0.28 Ang.
PLAT242_ALERT_2_B Low 'MainMol' Ueq as Compared to Neighbors of C11 Check

---

Alert level C

RINTA01_ALERT_3_C The value of Rint is greater than 0.12
Rint given 0.176
PLAT020_ALERT_3_C The Value of Rint is Greater Than 0.12 .... 0.176 Report
PLAT220_ALERT_2_C NonSolvent Resd 1 C Ueq(max)/Ueq(min) Range 4.6 Ratio
PLAT222_ALERT_3_C NonSolvent Resd 1 H Uiso(max)/Uiso(min) Range 5.1 Ratio
PLAT234_ALERT_4_C Large Hirshfeld Difference C11 --C12 . 0.18 Ang.
PLAT242_ALERT_2_C Low 'MainMol' Ueq as Compared to Neighbors of O5 Check
PLAT341_ALERT_3_C Low Bond Precision on C-C Bonds .... 0.01364 Ang.
PLAT414_ALERT_2_C Short Intra D-H..H-X H5 ..H26A 1.98 Ang.
x,y,z = 1_555 Check
PLAT906_ALERT_3_C Large K Value in the Analysis of Variance ...... 3.890 Check
PLAT911_ALERT_3_C Missing FCF Refl Between Thmin & STh/L= 0.572 11 Report
PLAT977_ALERT_2_C Check Negative Difference Density on H26C -0.37 eA-3

Alert level G

PLAT002_ALERT_2_G Number of Distance or Angle Restraints on AtSite 4 Note
PLAT003_ALERT_2_G Number of Uiso or Uij Restrained non-H Atoms ... 3 Report
PLAT154_ALERT_1_G The s.u.'s on the Cell Angles are Equal ..(Note) 0.019 Degree
PLAT172_ALERT_4_G The CIF-Embedded .res File Contains DFIX Records 1 Report
PLAT173_ALERT_4_G The CIF-Embedded .res File Contains DANG Records 2 Report
PLAT177_ALERT_4_G The CIF-Embedded .res File Contains DELU Records 1 Report
PLAT186_ALERT_4_G The CIF-Embedded .res File Contains ISOR Records 2 Report
PLAT199_ALERT_1_G Reported _cell_measurement_temperature ..... (K) 273 Check
PLAT200_ALERT_1_G Reported _diffrn_ambient_temperature ..... (K) 273 Check
PLAT605_ALERT_4_G Largest Solvent Accessible VOID in the Structure 24 A**3
PLAT794_ALERT_5_G Tentative Bond Valency for Cu1 (II) . 2.34 Info
PLAT794_ALERT_5_G Tentative Bond Valency for Cu2 (II) . 2.22 Info
PLAT860_ALERT_3_G Number of Least-Squares Restraints .... 15 Note
PLAT910_ALERT_3_G Missing # of FCF Reflection(s) Below Theta(Min). 4 Note
PLAT978_ALERT_2_G Number C-C Bonds with Positive Residual Density. 0 Info

---

0 **ALERT level A** = Most likely a serious problem - resolve or explain
4 **ALERT level B** = A potentially serious problem, consider carefully
11 **ALERT level C** = Check. Ensure it is not caused by an omission or oversight
15 **ALERT level G** = General information/check it is not something unexpected

3 ALERT type 1 CIF construction/syntax error, inconsistent or missing data
8 ALERT type 2 Indicator that the structure model may be wrong or deficient
9 ALERT type 3 Indicator that the structure quality may be low
8 ALERT type 4 Improvement, methodology, query or suggestion
2 ALERT type 5 Informative message, check

It is advisable to attempt to resolve as many as possible of the alerts in all categories. Often the minor alerts point to easily fixed oversights, errors and omissions in your CIF or refinement strategy, so attention to these fine details can be worthwhile. In order to resolve some of the more serious problems it may be necessary to carry out additional measurements or structure refinements. However, the purpose of your study may justify the reported deviations and the more serious of these should normally be commented upon in the discussion or experimental section of a paper or in the "special_details" fields of the CIF. checkCIF was carefully designed to identify outliers and unusual parameters, but every test has its limitations and alerts that are not important in a particular case may appear. Conversely, the absence of alerts does not guarantee there are no aspects of the results needing attention. It is up to the individual to critically assess their own results and, if necessary, seek expert advice.

Publication of your CIF in IUCr journals

A basic structural check has been run on your CIF. These basic checks will be run on all CIFs submitted for publication in IUCr journals (*Acta Crystallographica*, *Journal of Applied Crystallography*, *Journal of Synchrotron Radiation*); however, if you intend to submit to *Acta Crystallographica Section C* or *E* or *IUCrData*, you should make sure that full publication checks are run on the final version of your CIF prior to submission.

Publication of your CIF in other journals

Please refer to the *Notes for Authors* of the relevant journal for any special instructions relating to CIF submission.

PLATON version of 05/12/2020; check.def file version of 05/12/2020

Datablock qq - ellipsoid plot

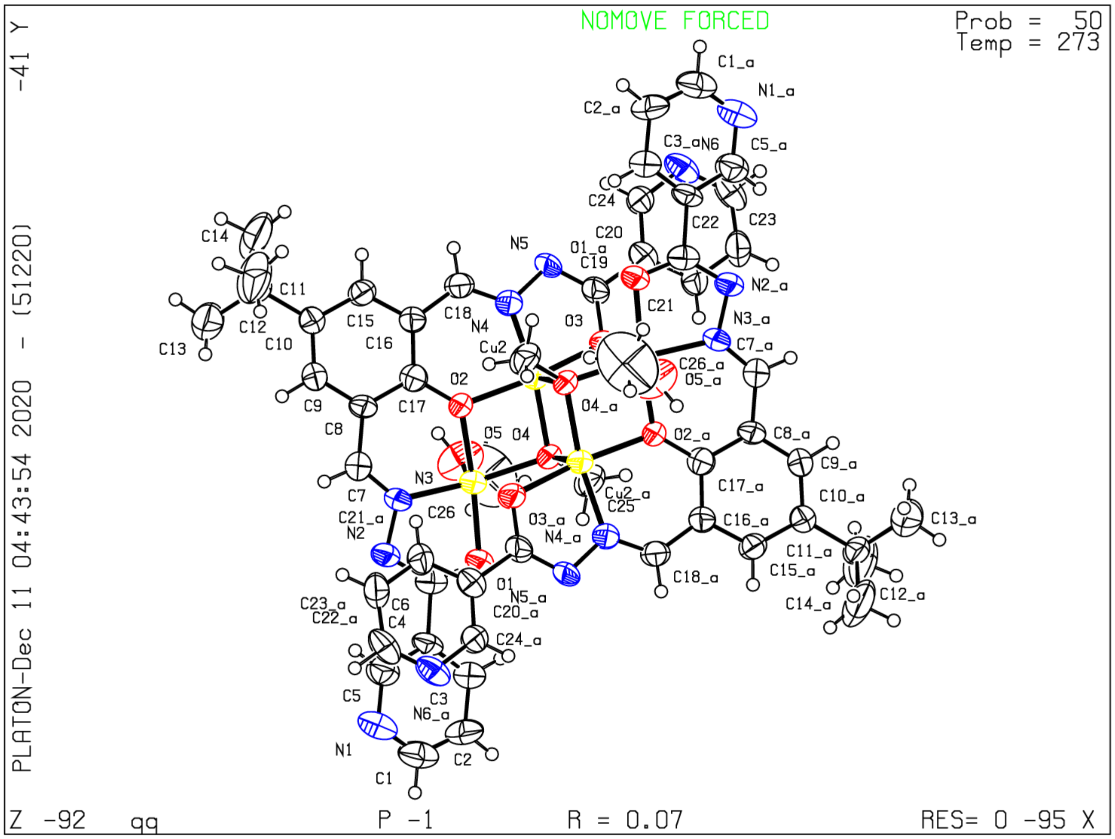

Supplement: Supplementary file 1 [file molecules-26-00109-s001.zip › cif/complex 1-checkcif.pdf]
