# Supplementary material for: Self-Assembly by Tridentate or Bidentate Ligand: Synthesis and Vapor Adsorption Properties of Cu(II), Zn(II), Hg(II) and Cd(II) Complexes Derived from a Bis(pyridylhydrazone) Compound
Source: Molecules. 2020 Dec 29;26(1):109. doi: 10.3390/molecules26010109 (PMC7795747; doi:10.3390/molecules26010109)

checkCIF/PLATON report

You have not supplied any structure factors. As a result the full set of tests cannot be run.

THIS REPORT IS FOR GUIDANCE ONLY. IF USED AS PART OF A REVIEW PROCEDURE FOR PUBLICATION, IT SHOULD NOT REPLACE THE EXPERTISE OF AN EXPERIENCED CRYSTALLOGRAPHIC REFEREE.

No syntax errors found. CIF dictionary Interpreting this report

Datablock: tt

Bond precision: C-C = 0.0052 A Wavelength=0.71073

Cell:	a=9.019(5)	b=12.473(7)	c=15.45(1)
	alpha=93.56(2)	beta=103.431(19)	gamma=105.544(18)
Temperature:	273 K		

	Calculated	Reported
Volume	1614.3(17)	1614.3(16)
Space group	P -1	P -1
Hall group	-P 1	-P 1
Moiety formula	C48 H48 Cl4 N12 O6 Zn2 [+ solvent]	C48 H48 Cl4 N12 O6 Zn2
Sum formula	C48 H48 Cl4 N12 O6 Zn2 [+ solvent]	C48 H48 Cl4 N12 O6 Zn2
Mr	1161.56	1161.52
Dx,g cm-3	1.195	1.195
Z	1	1
Mu (mm-1)	0.957	0.957
F000	596.0	596.0
F000’	597.33	
h,k,lmax	10,14,18	10,14,18
Nref	5692	5680
Tmin,Tmax		
Tmin’		

Correction method= Not given

Data completeness= 0.998 Theta(max)= 24.997

R(reflections)= 0.0459(4404) wR2(reflections)= 0.1397(5680)

S = 1.088 Npar= 329

The following ALERTS were generated. Each ALERT has the format
test-name_ALERT_alert-type_alert-level.
Click on the hyperlinks for more details of the test.

Alert level C

```
PLAT053_ALERT_1_C Minimum Crystal Dimension Missing (or Error) ...     Please Check
PLAT054_ALERT_1_C Medium  Crystal Dimension Missing (or Error) ...     Please Check
PLAT055_ALERT_1_C Maximum Crystal Dimension Missing (or Error) ...     Please Check
PLAT242_ALERT_2_C Low    'MainMol' Ueq as Compared to Neighbors of        C11 Check
PLAT420_ALERT_2_C D-H Without Acceptor       N5       --H5       .     Please Check
```

Alert level G

```
PLAT007_ALERT_5_G Number of Unrefined Donor-H Atoms ..............          3 Report
PLAT199_ALERT_1_G Reported _cell_measurement_temperature ..... (K)        273 Check
PLAT200_ALERT_1_G Reported   _diffrn_ambient_temperature ..... (K)        273 Check
PLAT606_ALERT_4_G Solvent Accessible VOID(S) in Structure ........          ! Info
PLAT794_ALERT_5_G Tentative Bond Valency for Zn1       (II)      .       1.92 Info
```

---

0 **ALERT level A** = Most likely a serious problem - resolve or explain
0 **ALERT level B** = A potentially serious problem, consider carefully
5 **ALERT level C** = Check. Ensure it is not caused by an omission or oversight
5 **ALERT level G** = General information/check it is not something unexpected

5 ALERT type 1 CIF construction/syntax error, inconsistent or missing data
2 ALERT type 2 Indicator that the structure model may be wrong or deficient
0 ALERT type 3 Indicator that the structure quality may be low
1 ALERT type 4 Improvement, methodology, query or suggestion
2 ALERT type 5 Informative message, check

It is advisable to attempt to resolve as many as possible of the alerts in all categories. Often the minor alerts point to easily fixed oversights, errors and omissions in your CIF or refinement strategy, so attention to these fine details can be worthwhile. In order to resolve some of the more serious problems it may be necessary to carry out additional measurements or structure refinements. However, the purpose of your study may justify the reported deviations and the more serious of these should normally be commented upon in the discussion or experimental section of a paper or in the "special_details" fields of the CIF. checkCIF was carefully designed to identify outliers and unusual parameters, but every test has its limitations and alerts that are not important in a particular case may appear. Conversely, the absence of alerts does not guarantee there are no aspects of the results needing attention. It is up to the individual to critically assess their own results and, if necessary, seek expert advice.

Publication of your CIF in IUCr journals

A basic structural check has been run on your CIF. These basic checks will be run on all CIFs submitted for publication in IUCr journals (*Acta Crystallographica*, *Journal of Applied Crystallography*, *Journal of Synchrotron Radiation*); however, if you intend to submit to *Acta Crystallographica Section C* or *E* or *IUCrData*, you should make sure that full publication checks are run on the final version of your CIF prior to submission.

Publication of your CIF in other journals

Please refer to the *Notes for Authors* of the relevant journal for any special instructions relating to CIF submission.

PLATON version of 18/09/2020; check.def file version of 20/08/2020

Datablock tt - ellipsoid plot

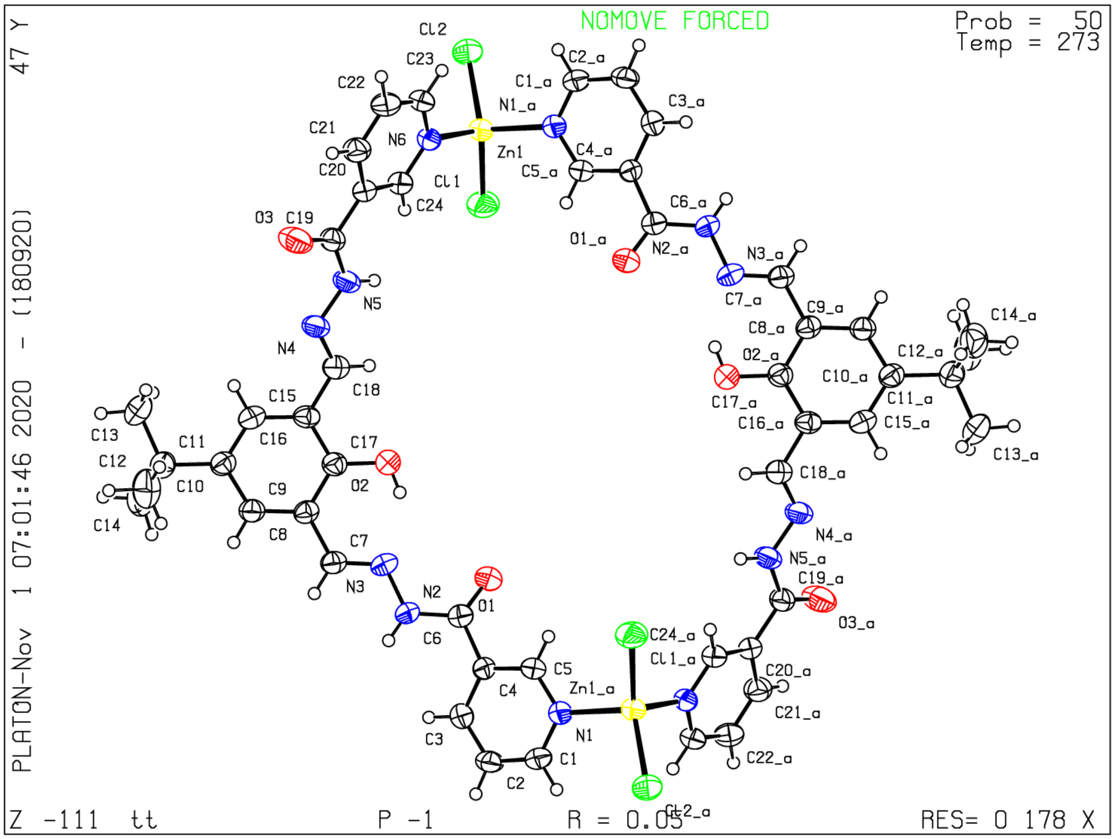

Supplement: Supplementary file 1 [file molecules-26-00109-s001.zip › cif/complex 2-checkcif.pdf]
