# Supplementary material for: Self-Assembly by Tridentate or Bidentate Ligand: Synthesis and Vapor Adsorption Properties of Cu(II), Zn(II), Hg(II) and Cd(II) Complexes Derived from a Bis(pyridylhydrazone) Compound
Source: Molecules. 2020 Dec 29;26(1):109. doi: 10.3390/molecules26010109 (PMC7795747; doi:10.3390/molecules26010109)
Supplement: Supplementary file 1 [file molecules-26-00109-s001.zip › Supplementary Materials.pdf]

Supplementary Materials

Self-assembly by tridentate or bidentate ligand: synthesis and vapor adsorption properties of Cu(II), Zn(II), Hg(II) and Cd(II) complexes derived from a bis(pyridylhydrazone) compound

Hong-Juan Liu, Rui Yi, Dong-Mei Chen, Chao Huang* and Bi-Xue Zhu*

Key Laboratory of Macrocyclic and Supramolecular Chemistry of Guizhou Province, Guizhou University, Guiyang 550025, China

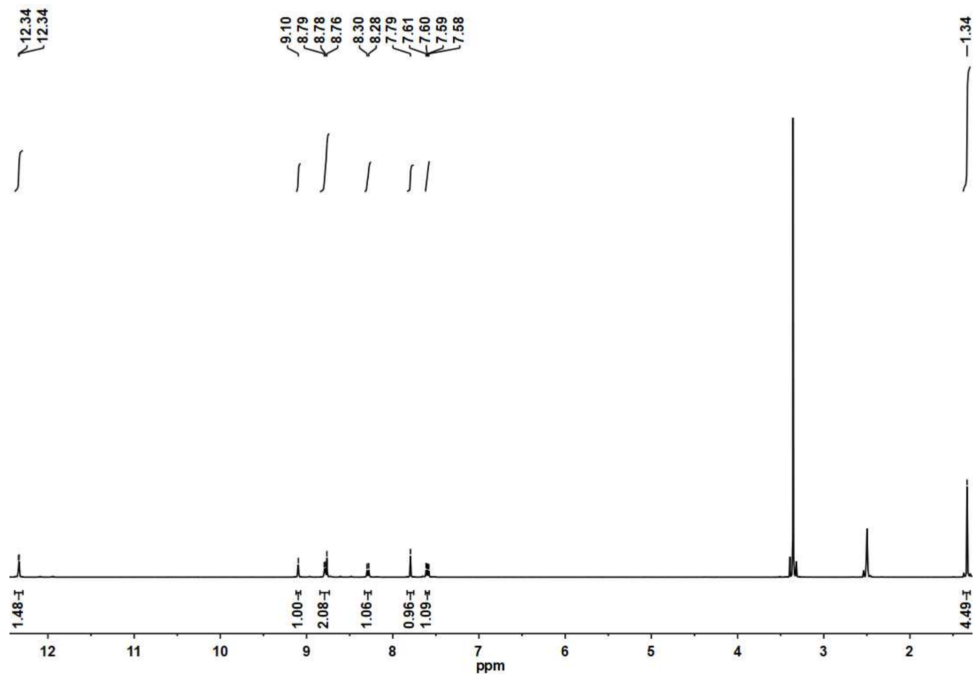

Figure. S1 ¹H NMR for **L**

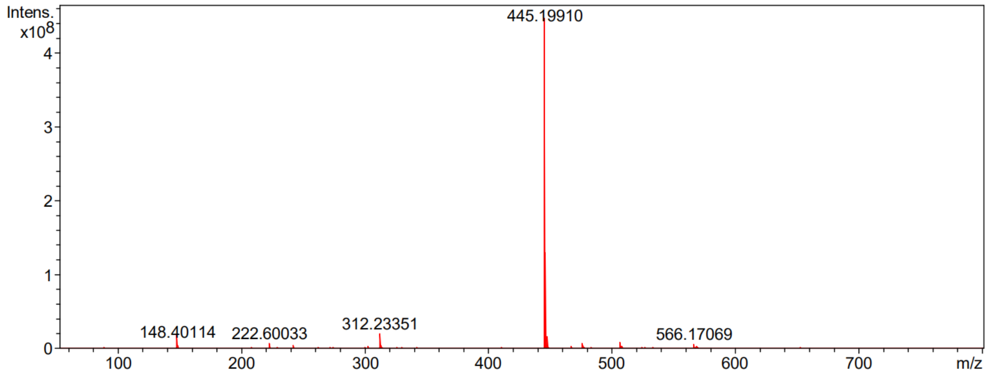

Figure. S2 MS for **L**

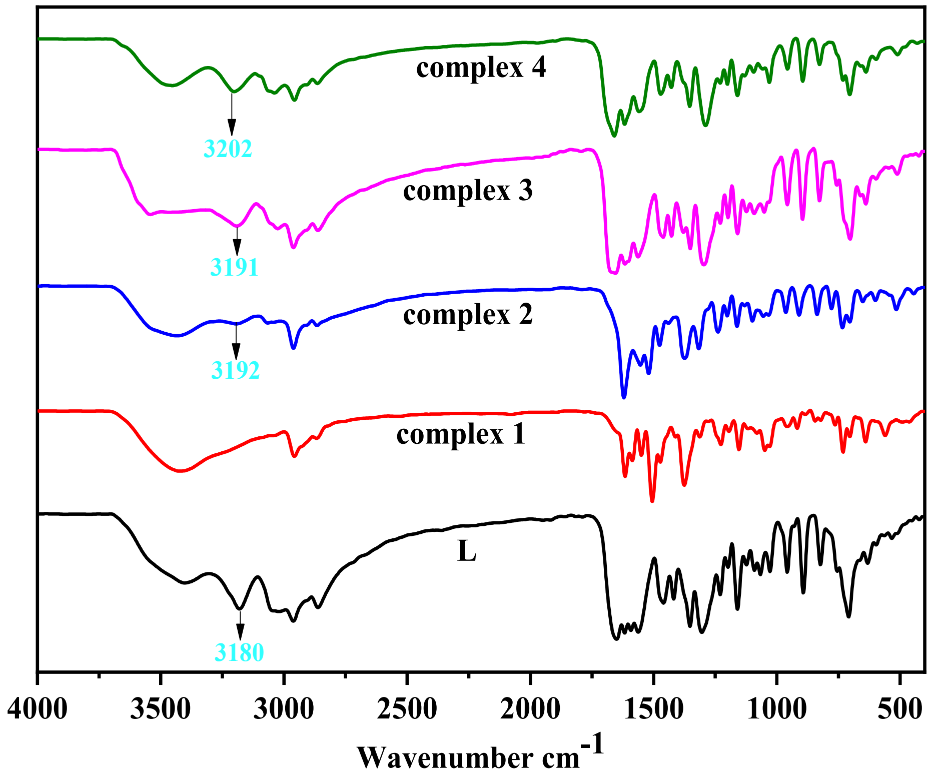

Figure. S3 IR spectra for **L**, complex **1**, complex **2**, complex **3** and complex **4**

Table S1 Selected bond distances (Å) and angles (°) for complexes **1**, **2**, **3** and **4**

Complex **1**					
Cu1−Cu2	2.957(2)	Cu1−O4	1.951(5)	Cu1−O2	1.935(5)
Cu1−O1	1.945(5)	Cu1−N3	1.914(7)	Cu1−O5	2.388(7)
Cu2#1−O4	2.377(6)	Cu2−O4	1.959(5)	Cu2−O2	1.952(5)
Cu2−O3	1.941(6)	Cu2−N4	1.918(7)		
N3−Cu1−O1	83.0(3)	N3−Cu1−O5	97.0(3)	O2−Cu2−O4#1	97.0(2)
O1−Cu1−O4	103.9(2)	O1−Cu1−O5	98.2(3)	O4−Cu2−O4#1	85.9(2)
N3−Cu1−O4	166.5(3)	N3−Cu1−O2	91.1(3)	O3−Cu2−O4#1	91.8(2)
O3−Cu2−O2	170.2(2)	O2−Cu1−O1	171.9(2)	N4−Cu2−O4	170.2(3)
N4#1−Cu2#1−O4#1	100.5(2)	N4−Cu2−O2	91.7(3)	N4−Cu2−O3	82.4(3)
O2−Cu1−O4	80.8(2)	O4−Cu1−O5	93.4(3)	O2−Cu1−O5	88.0(3)
Complex **2**					
Zn1−Cl2	2.2070(16)	Zn1−Cl1	2.2264(16)	Zn1−N6	2.060(3)#2
Zn1−N1	2.079(3)				
Cl2−Zn1−Cl1	123.80(5)	N6#2−Zn1−Cl2	108.10(8)	N6#2−Zn1−Cl1	108.21(9)
N6#2−Zn1−N1	104.21(12)	N1−Zn1−Cl2	106.38(9)	N1−Zn1−Cl1	104.46(8)
Complex **3**					
N6#3−Hg1	2.389(4)	N1−Hg1	2.412(4)	Hg1−N6#3	2.389(4)
Hg1−Br1	2.4718(6)	Hg1−Br2	2.4703(6)		
N6#3−Hg1−N1	95.64(15)	N6−Hg1−Br1	103.21(10)	N6−Hg1−Br2	103.48(10)
N1−Hg1−Br1	98.92(10)	N1−Hg1−Br2	101.49(10)	Br2−Hg1−Br1	144.37(3)
Complex **4**					
N1−Cd1	2.418(4)	N6#4−Cd1	2.420(4)	Cd1−Cl1	2.5693(11)
Cd1−Cl1#4	2.5693(11)				
N1−Cd1−N1#5	180.0(2)	N1−Cd1−N6	90.98(12)	N1#5−Cd1−N6#6	89.02(12)
N1#5−Cd1−N6	90.98(12)	N1−Cd1−N6#6	89.02(12)	N1#5−Cd1−Cl1	89.82(9)
N1#5−Cd1−Cl1#4	90.18(9)	N1−Cd1−Cl1	89.82(9)	N1−Cd1−Cl1#4	90.18(9)
N1#5−Cd1−N6#6	180.0	N6#6−Cd1−Cl1	91.05(9)	N6#6−Cd1−Cl1#4	91.05(9)
N6−Cd1−Cl1	88.95(9)	N6−Cd1−Cl1#4	88.95(9)	Cl1−Cd1−Cl1#4	180.0

Symmetry codes: #1 1-*x*, 1-*y*, 1-*z*; #2 *x*, *y*, -1+*z*; #3 *x*, *y*, -1+*z*; #4 1-*x*, -*y*, 1-*z*; #5 -*x*, -*y*, 2-*z*; #6 -1+*x*, *y*, 1+*z*

Table S2 Hydrogen bonding distances (Å) and angles (°) for complexes **1-4**

Complex **1**	D−H···A	D−H(Å)	H···A(Å)	D···A(Å)	∠DHA
	O5−H5A···N6	0.87(10)	2.00(10)	2.767(12)	146(8)
Complex **2**	O2−H2···N3	0.82	1.89	2.610(4)	146
	N2−H2A···O3	0.86	2.05	2.874(4)	161
Complex **3**	N2−H2A···O3	0.87	2.01	2.836(6)	158
	O2−H2B···N3	0.83	1.87	2.589(6)	145
Complex **4**	N2−H2A···O3	0.87	2.02	2.861(5)	162
	O2−H2B···N3	0.83	1.89	2.619(5)	146
	O4−H4···Cl1	0.83	2.30	3.112(4)	168
	N5−H5A···O4	0.87	1.99	2.818(6)	159
	O5−H5B···O1	0.83	1.90	2.726(10)	172
